# Supplementary material for: Coordination of two enhancers drives expression of olfactory trace amine-associated receptors
Source: Nat Commun. 2021 Jun 18;12:3798. doi: 10.1038/s41467-021-23823-4 (PMC8213717; doi:10.1038/s41467-021-23823-4)
Supplement: Supplementary file 1 — Supplementary Information [file 41467_2021_23823_MOESM1_ESM.pdf]

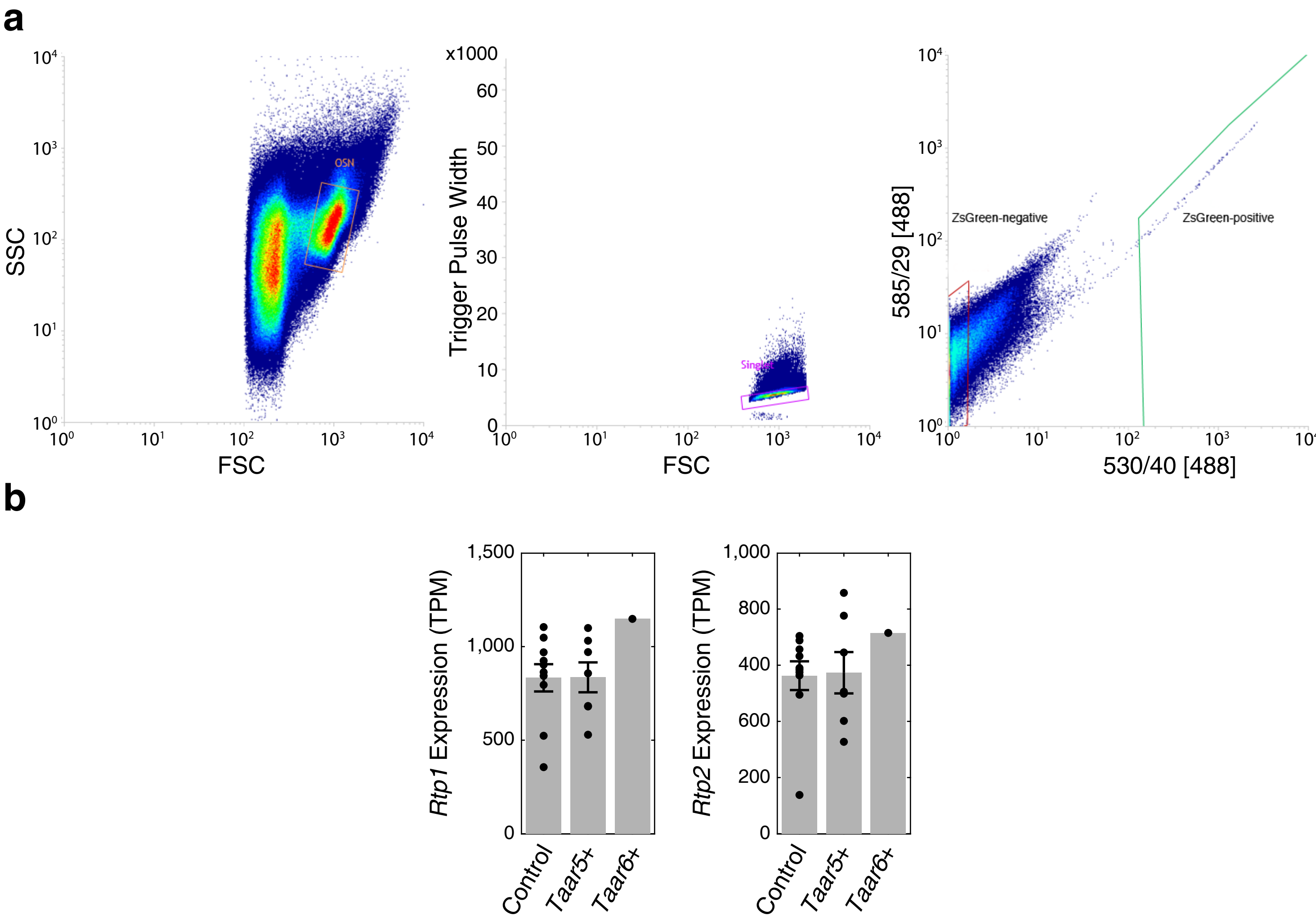

Supplementary figure 1

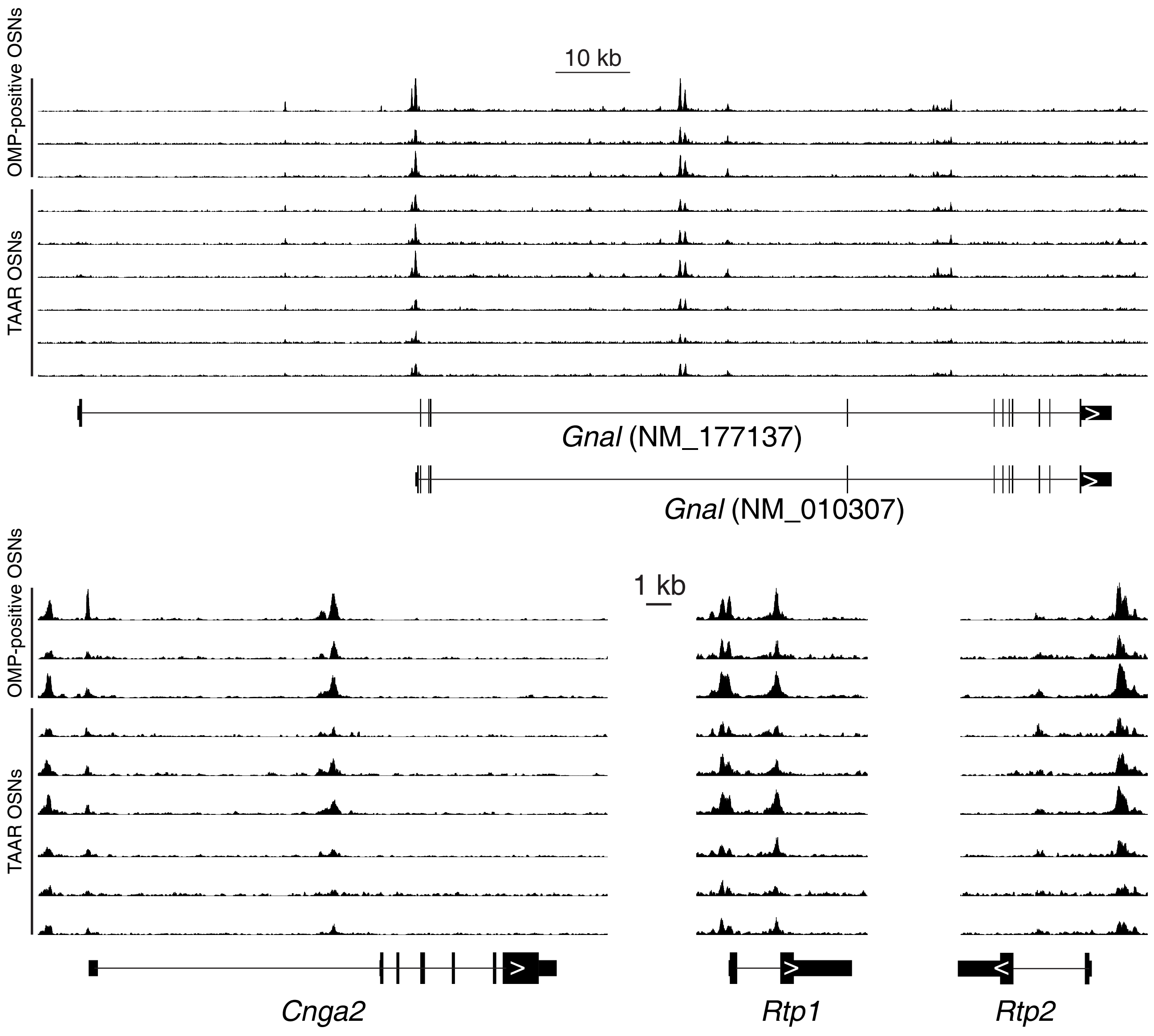

Supplementary figure 2

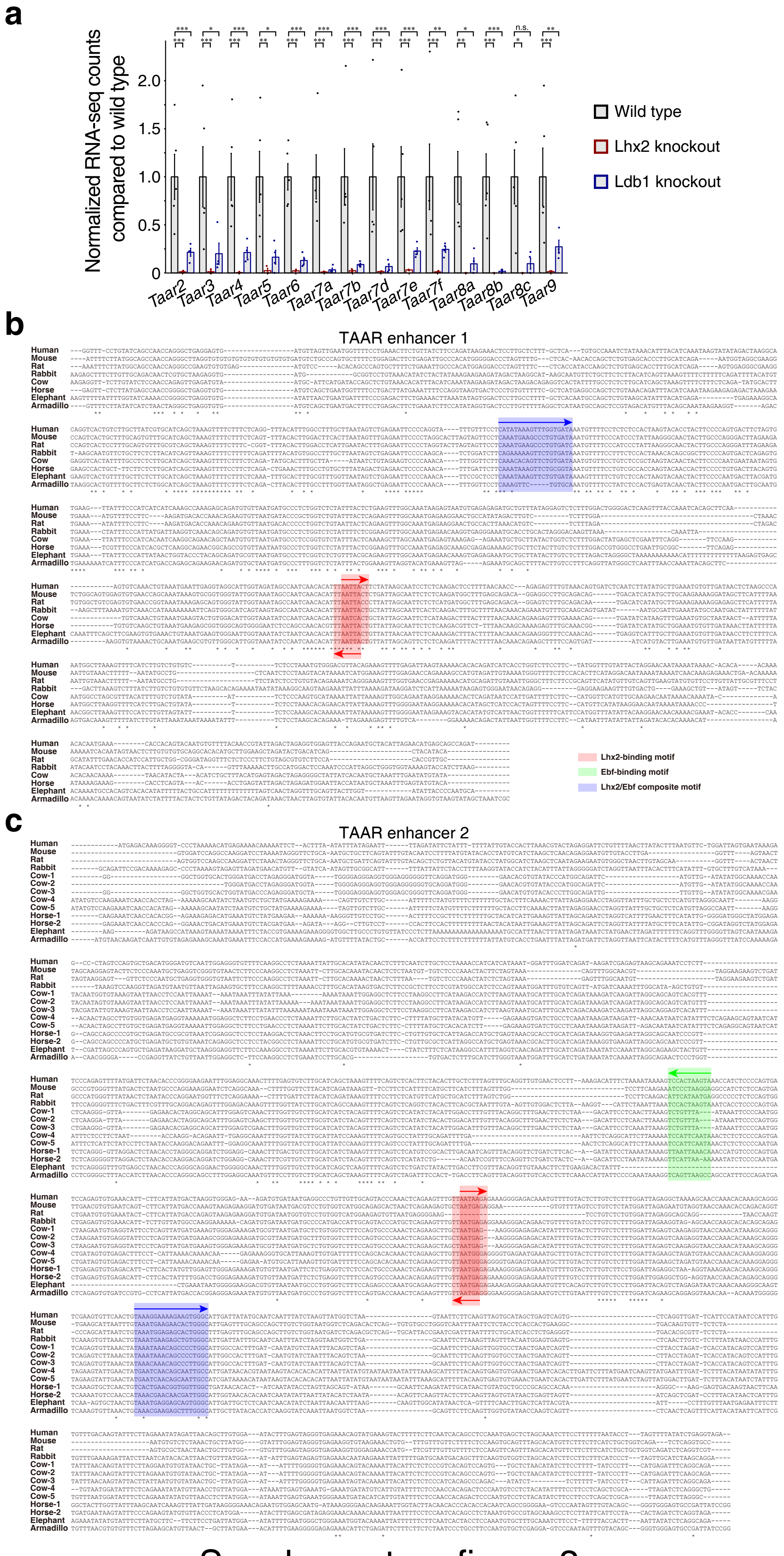

Supplementary figure 3

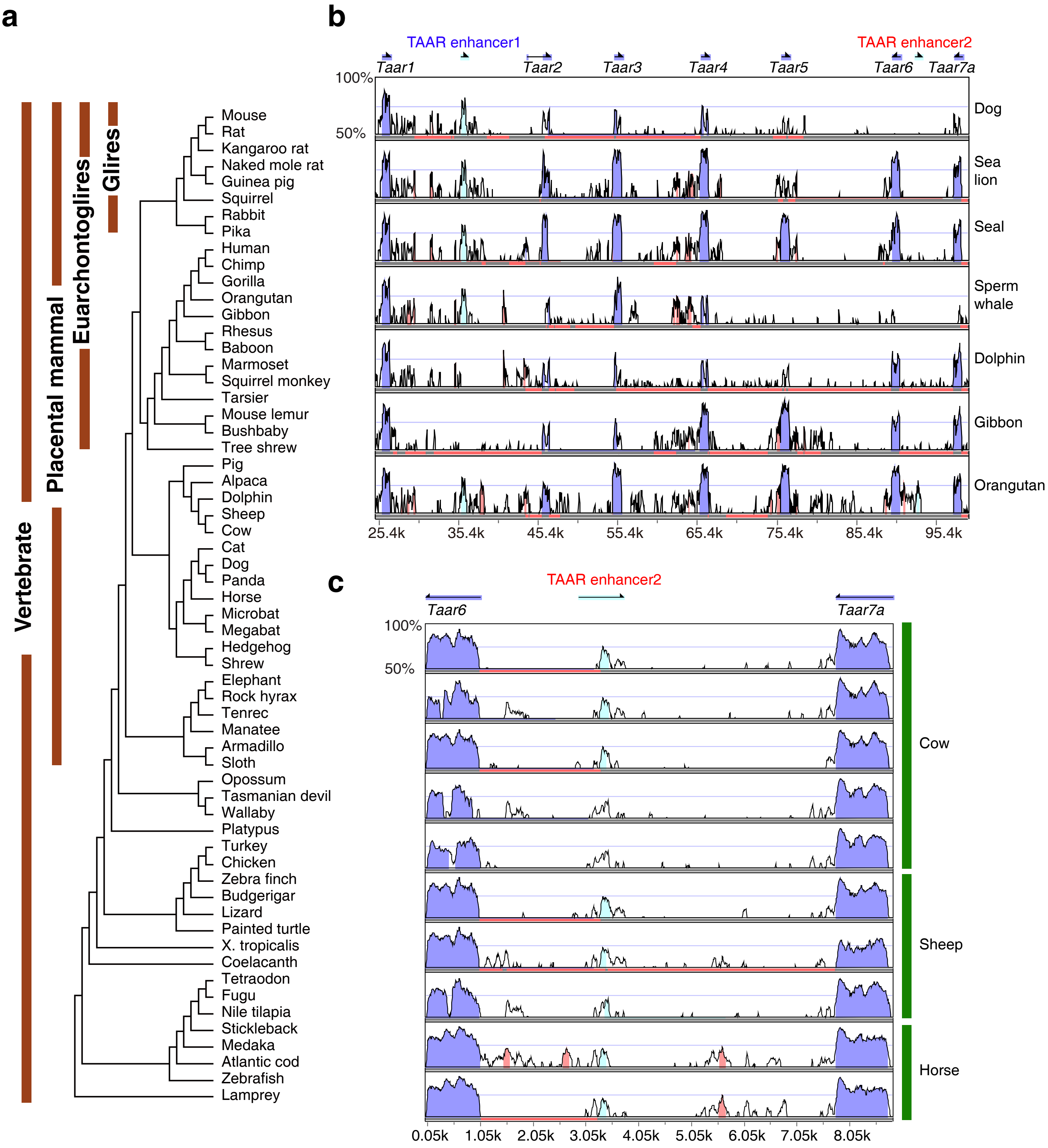

Supplementary figure 4

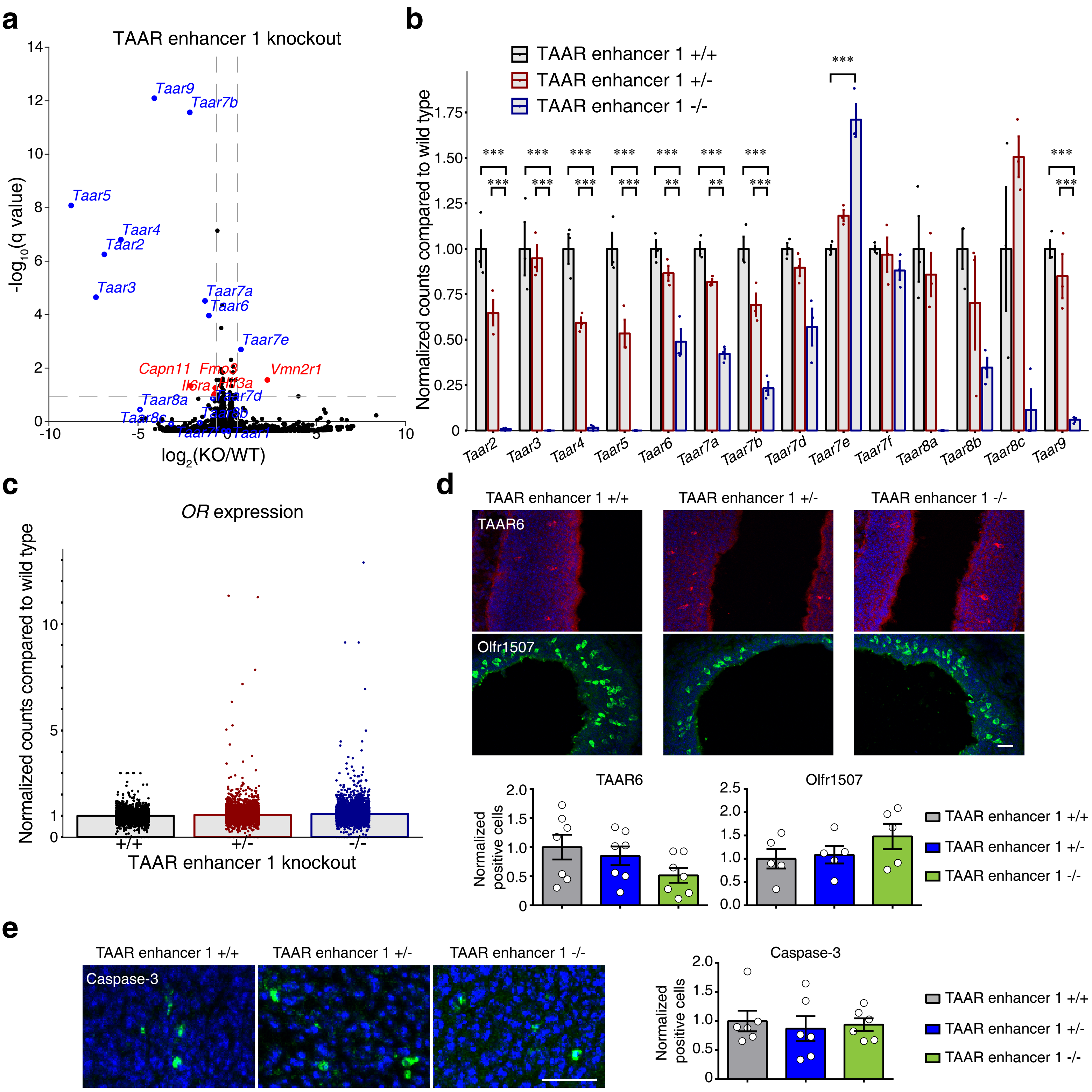

Supplementary figure 5

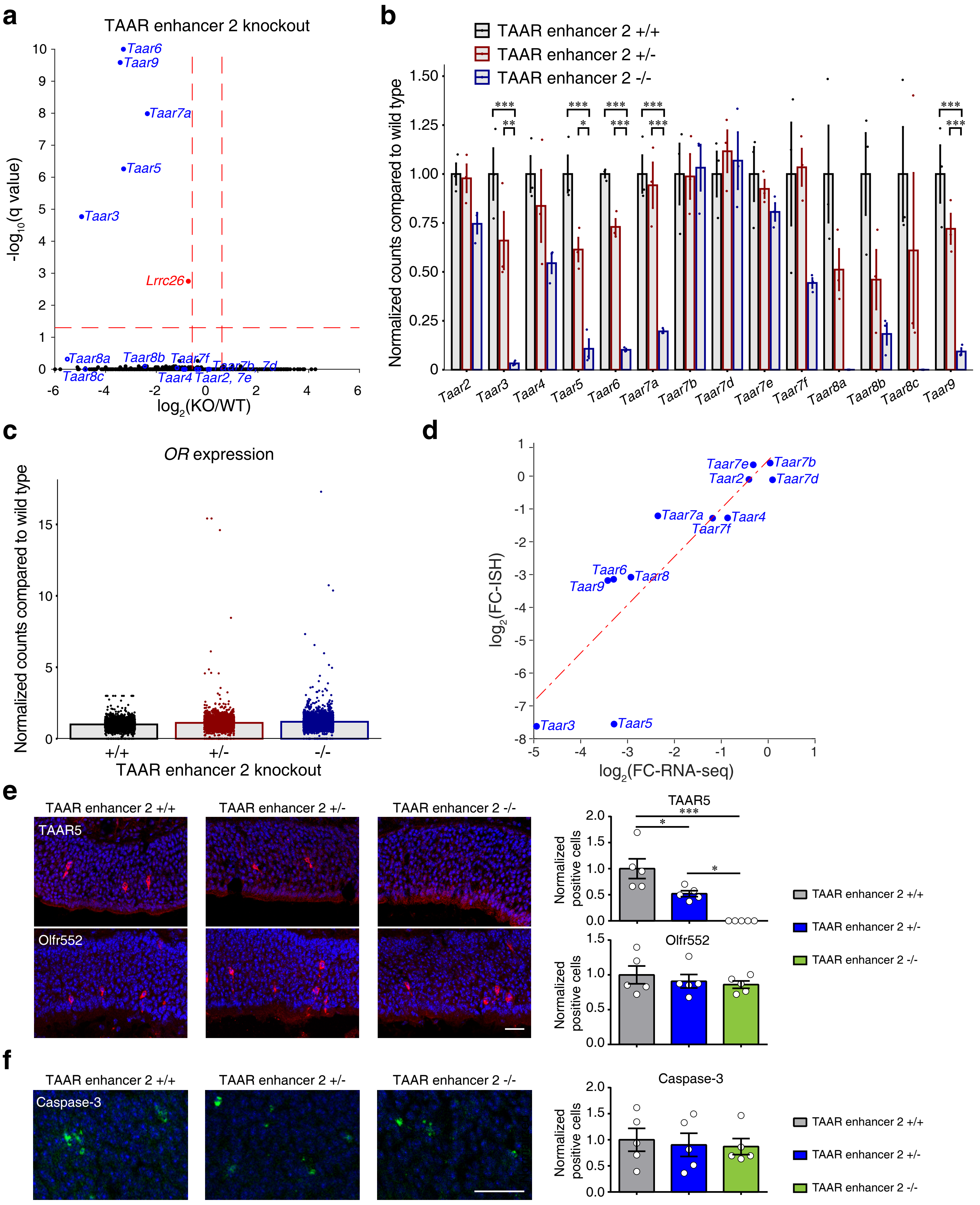

Supplementary figure 6

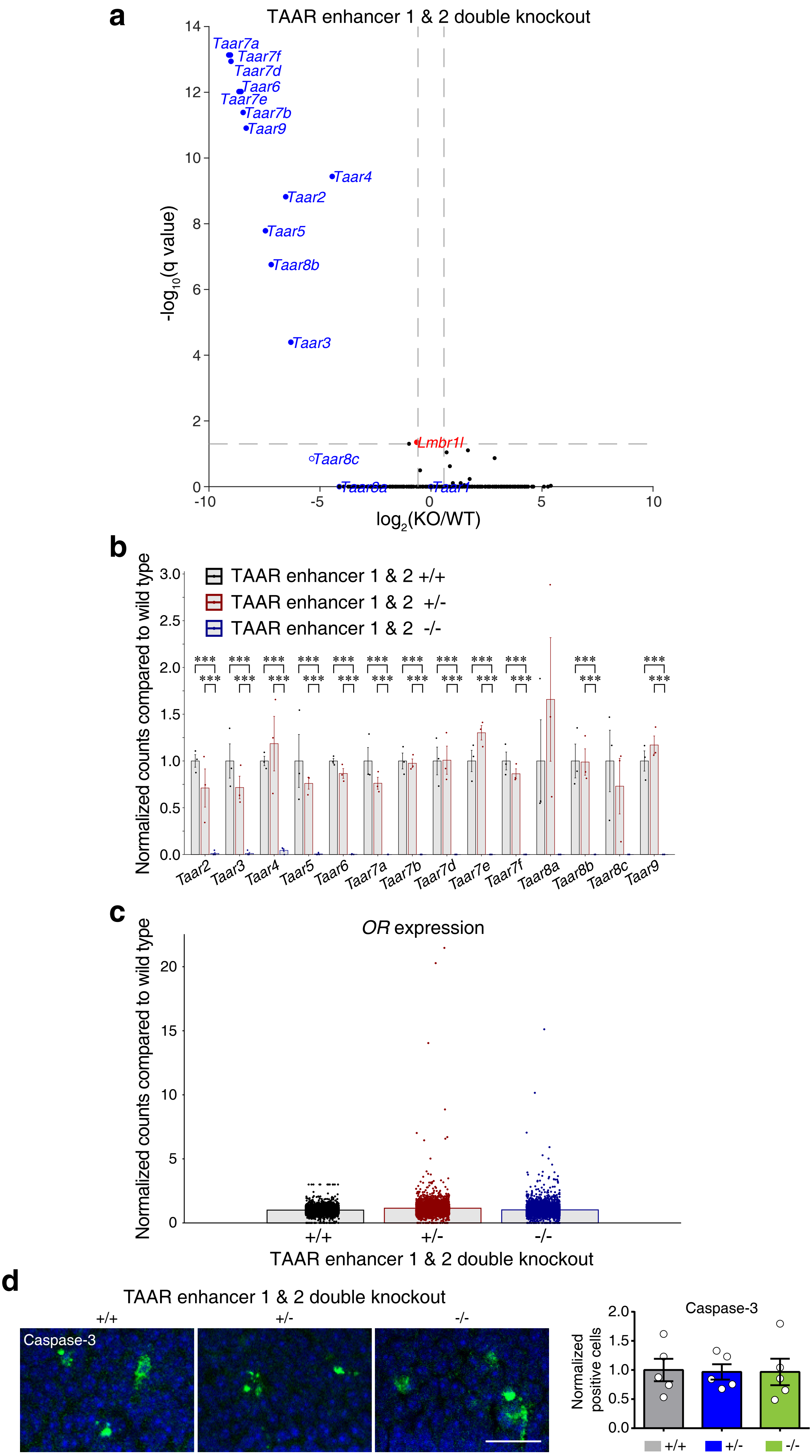

Supplementary figure 7

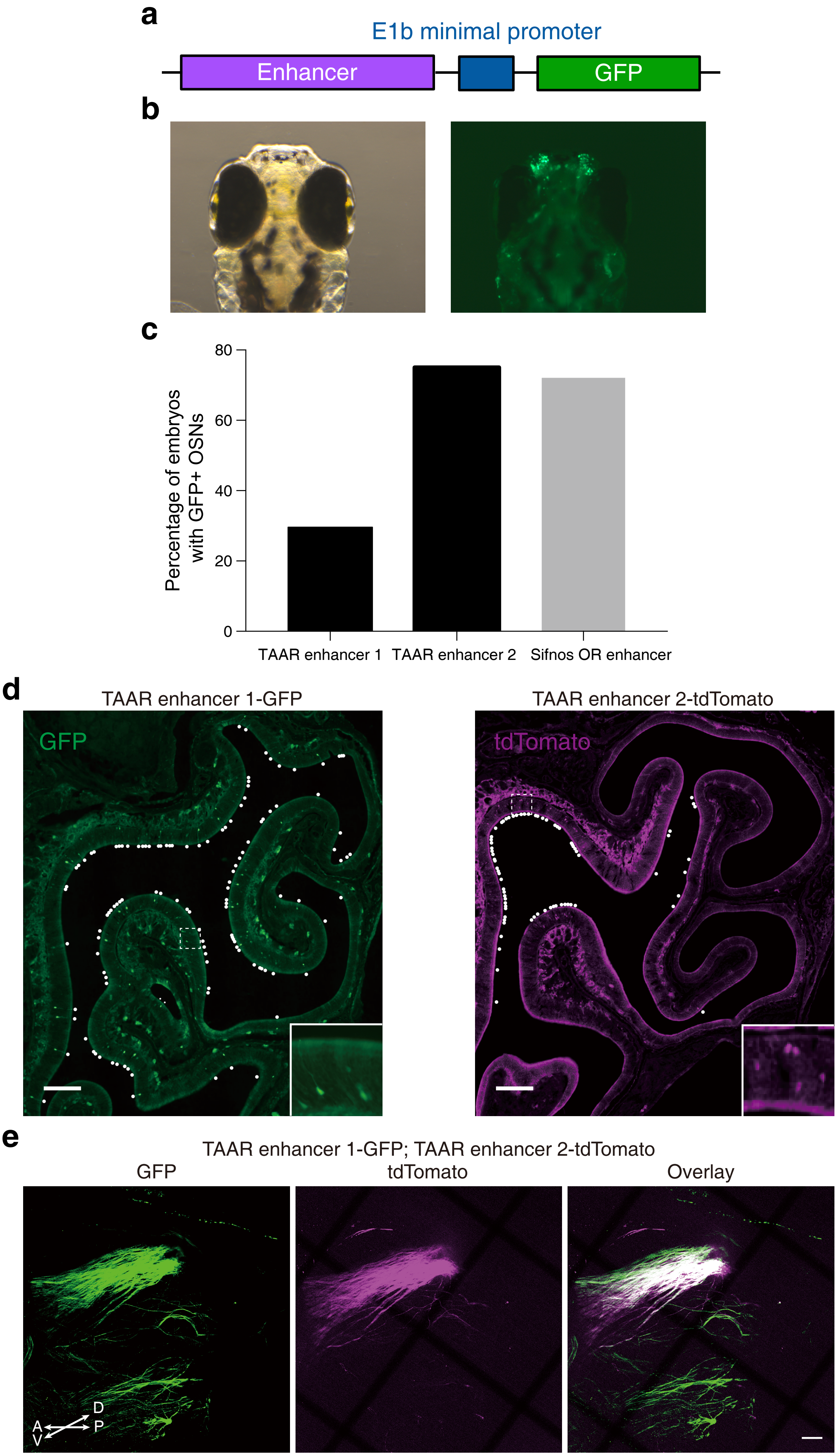

Supplementary figure 8

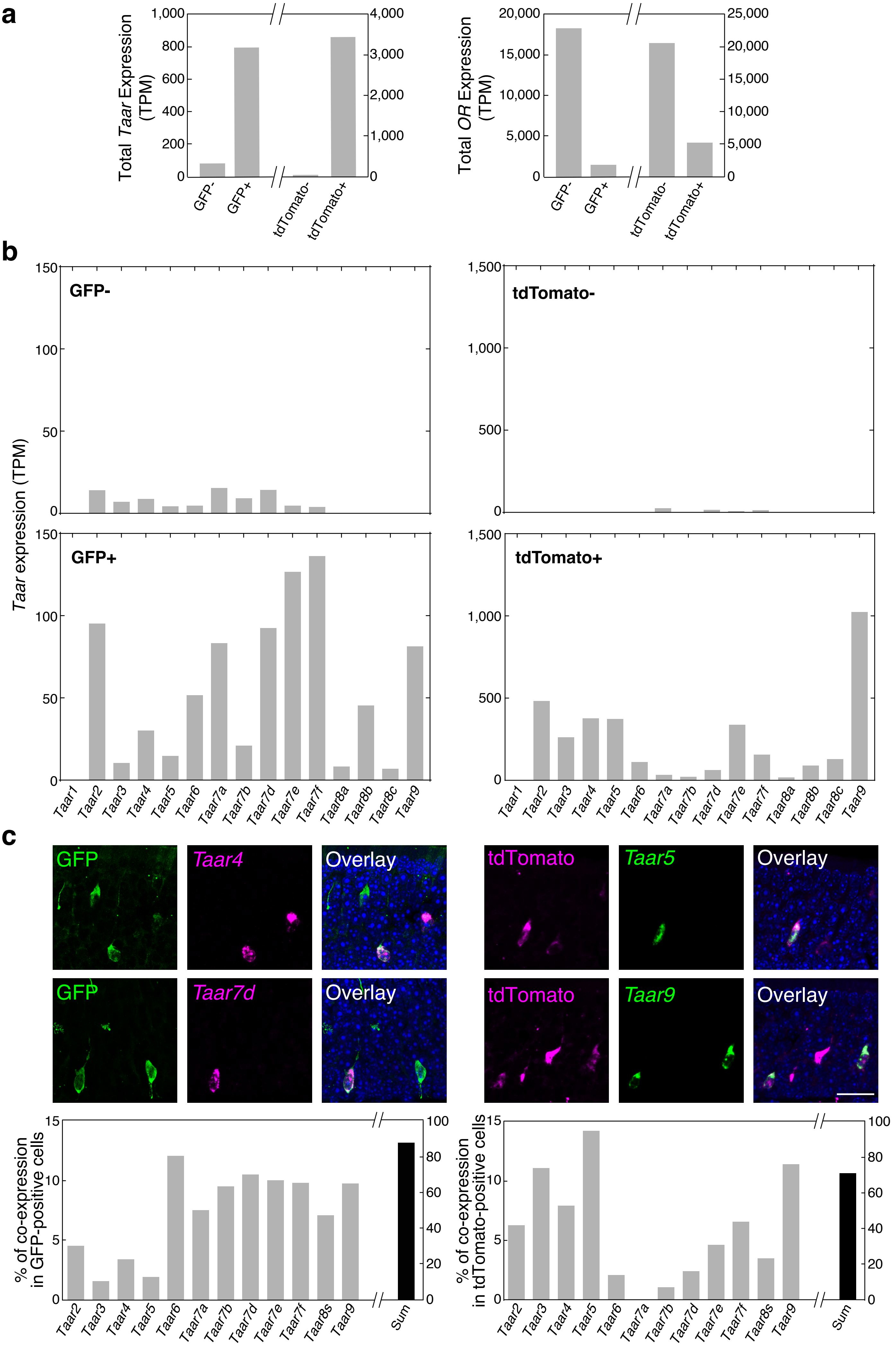

Supplementary figure 9

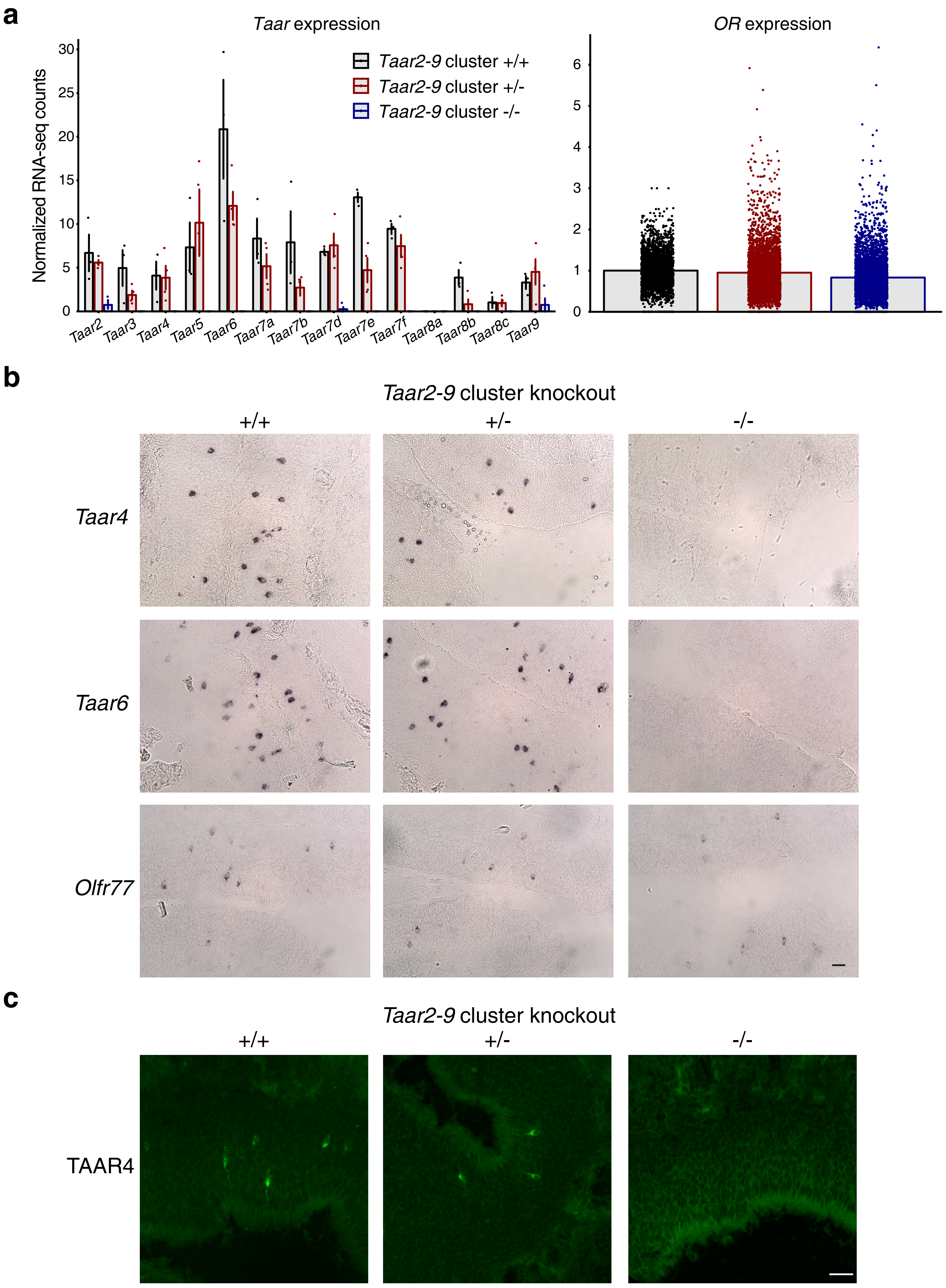

Supplementary figure 10

Supplementary Table 1. The information of extracted genome sequences from various mammalian species				
Species	Species	Genome assembly version	Genome coordinates	Genome regions
Mouse	Mus musculus	GRCm38.p4	chr10:23894688-24188961	Vnn1-Taar cluster-Stx7
			chr10:23920406-23941583	Taar1-Taar2
			chr10:23984609-23993481	Taar6-Taar7a
Human	Homo sapiens	GRCh38.p12	chr6:c132646026-132617150	Taar1-Taar2
			chr6:c132571359-132559024	Taar6-Taar7P
Chimpanzee	Pan troglodytes	Clint_PTRv2	chr6:c130003330-129974149	Taar1-Taar2P
			chr6:c129928126-129909449	Taar6-Taar8P
Orangutan	Pongo abelii	Susie_PABv2	chr6:c131193663-130936926	Vnn1-Taar cluster-Stx7
Gibbon	Nomascus leucogenys	Nleu_3.0	chr3:c119980447-119808147	Vnn1-Taar cluster-Stx7
Tarsier	Carlito syrichta	Tarsius_syrichta-2.0.1	NW_007247600.1:c380786-349157	Taar1-Taar2
			NW_007247600.1:c287550-257458	Taar6-Taar8
Rat	Rattus norvegicus	Rnor_6.0	chr1:c22596475-22578025	Taar1-Taar2
			chr1:c22537236-22528659	Taar6-Taar7a
Rabbit	Oryctolagus cuniculus	OryCun2.0	chr12:c122918612-122891431	Taar1-Taar2
			chr12:c122850827-122831128	Taar6-Taar6
Hedgehog	Erinaceus europaeus	EriEur2.0	NW_006804730.1:c662714-641243	Taar1-Taar2
			NW_006804730.1:c550178-516925	Taar6-Taar6
Pig	Sus scrofa	Sscrofa11.1	chr1:31037826-31074574	Taar1-Taar2
			chr1:31124661-31135694	Taar6-Taar7a
Cow	Bos taurus	ARS-UCD1.2	chr9:c70827654-70784406	Taar1-Taar2
			chr9:70591888-70609286	Taar6-Taar7aP
			chr9:c70554482-70533382	Taar8P-Taar7a
			chr9:c70711073-70693872	Taar6-Taar7a
			chr9:70618129-70633589	Taar8P-Taar7a
			chr9:c70534425-70518751	Taar7a-Taar7aP
Sheep	Ovis aries	Oar_rambouillet_v1.0	chr8:c64047965-64031195	Taar6-Taar7a
			chr8:c66398793-66380107	Taar6-Taar6P
			chr8:c64021497-64002370	Taar8P-Taar7a
Dolphin (Yangtze River do	Lipotes vexillifer	Lipotes_vexillifer_v1	NW_006784126.1:187583-324150	Vnn1-Taar cluster-Stx7
Sperm whale	Physeter catodon	ASM283717v2	chr10:30946785-31107969	Vnn1-Taar cluster-Stx7
			chr10:31012694-31027703	Taar1-Taar3P
Horse	Equus caballus	EquCab3.0	chr10:c80023263-80001724	Taar1-Taar2

			chr10:c79907708-79891692	Taar6-Taar7a
			chr10:79906594-79921740	Taar6-Taar7a
Cat	Felis catus	Felis_catus_9.0	chrB2:c122585612-122538799	Taar1-Taar2
			chrB2:122466294-122482960	Taar6-Taar7a
Dog	Canis lupus familiaris	CanFam3.1	chr1:c25544283-25704579	Vnn1-Taar cluster-Stx7
Sea lion (California sea lio	Zalophus californianus	mZalCal1.pri.v2	chr7:31727731-31962268	Vnn3-Taar cluster-Stx7
Seal (Hawaiian monk seal	Neomonachus schauinslan	ASM220157v1	NW_018734349.1:31564931-31783478	Vnn1-Taar cluster-Stx7
Big brown bat	Eptesicus fuscus	EptFus1.0	NW_007370651.1:55488823-55512018	Taar1-Taar2
			NW_007370651.1:55537933-55555994	Taar5-Taar9
Elephant	Loxodonta africana	Loxafr3.0	NW_003573420.1:27517488-27557889	Taar1-Taar2
			NW_003573420.1:27626820-27662685	Taar4-Taar7a
Armadillo	Dasypus novemcinctus	Dasnov3.0	NW_004498224.1:c223592-203709	begin-Taar2
			NW_004498224.1:c125945-91587	Taar6P-Taar7aP
Koala	Phascolarctos cinereus	phaCin_unsw_v4.1	NW_018343979.1:c15437684-15405259	Taar1-Taar2
			NW_018343979.1:c15183169-15157886	Taar6-Taar7a
Opossum	Monodelphis domestica	MonDom5	chr2:c407125000-407094705	Taar1-Taar2
			chr2:c406934871-406909558	Taar6-Taar7a
Platypus	Ornithorhynchus anatinus	Ornithorhynchus_anatinus-	NW_001794460.1:c1524776-1501778	Taar1-Taar2

Supplementary Table 2. *In situ* analysis in TAAR enhancer 1 knockout mice.

Receptor probe	n	WT (mean ± s.e.)		Heterozygous (mean ± s.e.)		Homozygous (mean ± s.e.)		One-way ANOVA p value
		Cell number	% of WT	Cell number	% of WT	Cell number	% of WT	
Taar2	5	21.0 ± 2.5	100.0 ± 12.1	13.4 ± 2.8	63.8 ± 13.4	0	0	< 0.0001
Taar3	15	22.5 ± 1.7	100.0 ± 7.6	15.1 ± 1.2	66.9 ± 5.2	0	0	< 0.0001
Taar4	7	24.4 ± 1.5	100.0 ± 6.2	20.4 ± 1.5	83.6 ± 6.3	1.6 ± 0.6	6.4 ± 2.3	< 0.0001
Taar5	7	13.3 ± 1.4	100.0 ± 10.9	9.1 ± 1.2	68.8 ± 8.8	0	0	< 0.0001
Taar6	10	98.6 ± 16.2	100.0 ± 16.4	82.5 ± 11.7	83.7 ± 11.9	32.0 ± 5.6	32.5 ± 5.6	0.0015
Taar7a	8	57.5 ± 10.7	100.0 ± 18.6	36.5 ± 3.6	63.5 ± 6.3	17.3 ± 2.2	30.0 ± 3.7	0.0013
Taar7b	8	66.1 ± 11.5	100.0 ± 17.3	45.0 ± 6.6	68.1 ± 10	13.9 ± 4.4	21.0 ± 6.7	0.0006
Taar7d	6	35.2 ± 6.3	100.0 ± 17.8	27.2 ± 2.8	77.3 ± 7.8	14.3 ± 2.8	40.8 ± 8.0	0.0117
Taar7e	5	30.0 ± 7.2	100.0 ± 23.9	30.6 ± 6.3	102.0 ± 20.9	45.4 ± 4.5	151.3 ± 14.9	0.1700
Taar7f	9	34.1 ± 4.0	100.0 ± 11.8	29.9 ± 4.4	87.6 ± 12.8	22.2 ± 2.1	65.1 ± 6.3	0.0846
Taar8s	5	45.8 ± 14.8	100.0 ± 32.3	47.2 ± 5.9	103.1 ± 12.9	9.8 ± 2.7	21.4 ± 6.0	0.0242
Taar9	9	29.0 ± 2.7	100.0 ± 9.2	23.6 ± 3.1	81.2 ± 10.8	2.2 ± 0.5	7.7 ± 1.6	< 0.0001
Olfr1507	4	181.0 ± 22.6	100.0 ± 12.5	174.0 ± 10.5	96.1 ± 5.8	218.3 ± 27.2	120.6 ± 15.0	0.3320
Olfr578	5	52.6 ± 7.4	100.0 ± 14.2	49.2 ± 5.2	93.5 ± 9.9	54.0 ± 5.9	102.7 ± 11.1	0.8575

Supplementary Table 3. Comparison of *in situ* and RNA-seq analyses in TAAR enhancer 1 knockout mice.

Receptor probe	Expression [% of WT, *in situ*] (mean ± s.e.)	n	p value	Expression [% of WT, RNA-seq]	n	p value
Taar2	0	5	1.5×10^{-6}	0.8	3	2.5×10^{-7}
Taar3	0	15	1.7×10^{-13}	0.6	3	1.0×10^{-7}
Taar4	6.4 ± 2.3	7	4.5×10^{-9}	1.6	3	7.0×10^{-8}
Taar5	0	7	8.7×10^{-7}	0.2	3	3.7×10^{-9}
Taar6	32.5 ± 5.6	10	1.1×10^{-3}	49.2	3	4.9×10^{-5}
Taar7a	30.0 ± 3.7	8	2.4×10^{-3}	42.3	3	1.4×10^{-5}
Taar7b	21.0 ± 6.7	8	8.0×10^{-4}	23.4	3	1.2×10^{-12}
Taar7d	40.8 ± 8.0	6	1.2×10^{-2}	57.0	3	6.1×10^{-2}
Taar7e	151.3 ± 14.9	5	0.106174	171.4	3	9.0×10^{-4}
Taar7f	65.1 ± 6.3	9	1.9×10^{-2}	88.0	3	0.999160
Taar8a				3.4	3	0.158900
Taar8b	21.4 ± 6.0	5	0.401288	34.8	3	0.495081
Taar8c				11.4	3	0.546081
Taar9	7.7 ± 1.6	9	3.2×10^{-8}	5.9	3	3.6×10^{-13}
Olfr1507	120.6 ± 15.0	4	0.332264	120.7	3	0.941256
Olfr578	102.7 ± 11.1	5	0.886369	118.2	3	0.9098223

Supplementary Table 4. *In situ* analysis in TAAR enhancer 2 knockout mice.

Receptor probe	n	WT (mean ± s.e.)		Heterozygous (mean ± s.e.)		Homozygous (mean ± s.e.)		One-way ANOVA p value
		Cell number	% of WT	Cell number	% of WT	Cell number	% of WT	
Taar2	7	18.7 ± 2.3	100.0 ± 12.2	18.4 ± 2.3	98.5 ± 12.6	17.4 ± 2.1	93.1 ± 11.1	0.9137
Taar3	4	24.3 ± 3.0	100.0 ± 12.3	13.3 ± 1.4	54.6 ± 5.7	0	0	< 0.0001
Taar4	8	34.6 ± 3.7	100.0 ± 10.8	29.4 ± 3.2	84.8 ± 9.2	14.3 ± 1.3	41.2 ± 3.6	0.0002
Taar5	4	13.3 ± 1.1	100.0 ± 8.4	6.8 ± 0.9	50.9 ± 6.4	0	0	< 0.0001
Taar6	5	98.8 ± 17.4	100.0 ± 17.6	69.8 ± 19.0	70.6 ± 19.3	11.0 ± 2.3	11.1 ± 2.4	0.0042
Taar7a	6	50.0 ± 4.4	100.0 ± 8.8	27.8 ± 4.7	55.7 ± 9.4	21.5 ± 4.8	43.0 ± 9.6	0.0014
Taar7b	8	57.6 ± 11.3	100.0 ± 19.5	63.0 ± 10.0	109.3 ± 17.3	76.0 ± 7.3	131.9 ± 12.7	0.4005
Taar7d	3	46.0 ± 13.2	100.0 ± 28.8	32.0 ± 4.0	69.6 ± 8.8	42.0 ± 13.8	91.3 ± 30.0	0.6817
Taar7e	5	30.2 ± 1.8	100.0 ± 5.9	28.4 ± 3.0	94.0 ± 9.9	38.4 ± 4.1	127.2 ± 13.6	0.0906
Taar7f	4	39.3 ± 0.8	100.0 ± 1.9	34.0 ± 3.6	86.6 ± 9.1	16.3 ± 2.5	41.4 ± 6.4	0.0003
Taar8s	4	42.8 ± 3.1	100.0 ± 7.3	38.0 ± 5.8	88.9 ± 13.7	5.0 ± 1.6	11.7 ± 3.7	0.0002
Taar9	10	28.0 ± 3.9	100.0 ± 14.1	22.1 ± 2.7	78.9 ± 9.8	3.0 ± 0.5	10.7 ± 1.9	< 0.0001
Olfr1507	4	117.8 ± 36.1	100.0 ± 30.7	157.8 ± 33.5	134.0 ± 28.4	91.0 ± 18.5	77.3 ± 15.7	0.3387
Olfr578	5	57.4 ± 7.6	100.0 ± 13.3	69.8 ± 4.1	121.6 ± 7.1	59.4 ± 4.9	103.5 ± 8.6	0.2981

Supplementary Table 5. Comparison of *in situ* and RNA-seq analyses in TAAR enhancer 2 knockout mice.

Receptor probe	Expression [% of WT, *in situ*] (mean ± s.e.)	n	p value	Expression [% of WT, RNA-seq]	n	p value
Taar2	93.1 ± 11.1	7	0.659573	75.2	3	0.999913
Taar3	0	4	1.8×10^{-4}	3.3	3	1.7×10^{-5}
Taar4	41.2 ± 3.6	8	1.4×10^{-4}	54.9	3	0.999913
Taar5	0	4	2.1×10^{-5}	10.2	3	5.5×10^{-7}
Taar6	11.1 ± 2.4	5	1.1×10^{-3}	10.2	3	3.0×10^{-30}
Taar7a	43.0 ± 9.6	6	1.4×10^{-3}	19.6	3	1.0×10^{-8}
Taar7b	131.9 ± 12.7	8	0.193209	102.9	3	0.999913
Taar7d	91.3 ± 30.0	3	0.844460	106.7	3	0.999913
Taar7e	127.2 ± 13.6	5	0.104065	80.2	3	0.999913
Taar7f	41.4 ± 6.4	4	1.2×10^{-4}	44.0	3	0.909372
Taar8a				2.2	3	0.481418
Taar8b	11.7 ± 3.7	4	3.7×10^{-5}	18.3	3	0.803564
Taar8c				3.6	3	0.999913
Taar9	10.7 ± 1.9	10	6.4×10^{-6}	9.3	3	2.6×10^{-10}
Olfr1507	77 ± 15.7	4	0.533878	128.7	3	0.999913
Olfr578	103 ± 8.6	5	0.831055	100.6	3	0.999913

Supplementary Table 6. *In situ* analysis in TAAR enhancer 1 & 2 knockout mice.

Receptor probe	n	WT (mean ± s.e.)		Heterozygous (mean ± s.e.)		Homozygous (mean ± s.e.)		One-way ANOVA p value
		Cell number	% of WT	Cell number	% of WT	Cell number	% of WT	
Taar2	5	23.2 ± 4.6	100 ± 19.9	14.2 ± 2.3	61.2 ± 10.0	0	0	0.0005
Taar3	10	24.3 ± 5.1	100 ± 21.1	15.3 ± 1.9	63.0 ± 7.8	0	0	< 0.0001
Taar4	5	38.6 ± 4.9	100 ± 12.8	25.6 ± 2.8	66.3 ± 7.4	0	0	< 0.0001
Taar5	5	26.8 ± 6.5	100 ± 24	20.8 ± 4.9	77.6 ± 18.0	0	0	0.0040
Taar6	10	113.1 ± 14.1	100 ± 12.5	74.6 ± 11.0	66.0 ± 9.7	0	0	< 0.0001
Taar7s	5	276.6 ± 48.8	100 ± 17.6	224.0 ± 39.6	81.0 ± 14.3	0	0	0.0004
Taar8s	10	113.4 ± 13.8	100 ± 12.2	87.2 ± 8.3	76.9 ± 7.3	0	0	< 0.0001
Taar9	10	34.2 ± 4.0	100 ± 11.7	32.6 ± 3.4	95.3 ± 10.0	0	0	< 0.0001
Olfr1507	8	195.0 ± 30.8	100 ± 15.8	215.3 ± 35.3	110.4 ± 18.1	228.9 ± 28.0	117.4 ± 14.4	0.7496
Olfr578	10	49.0 ± 4.7	100 ± 9.6	60.4 ± 5.7	123.3 ± 11.7	38.9 ± 5.8	79.4 ± 11.8	0.0318

Supplementary Table 7. Comparison of *in situ* and RNA-seq analyses in TAAR enhancer 1 & 2 double knockout mice.

Receptor probe	Expression [% of WT, *in situ*] (mean ± s.e.)	n	p value	Expression [% of WT, RNA-seq]	n	p value
Taar2	0	5	1.0×10^{-3}	1.1	≥ 3	1.5×10^{-9}
Taar3	0	10	1.7×10^{-4}	1.3	≥ 3	4.0×10^{-5}
Taar4	0	5	1.6×10^{-5}	4.6	≥ 3	3.6×10^{-10}
Taar5	0	5	3.3×10^{-3}	0.6	≥ 3	1.6×10^{-8}
Taar6	0	10	2.3×10^{-7}	0.3	≥ 3	9.5×10^{-13}
Taar7a				0.2	≥ 3	7.3×10^{-14}
Taar7b				0.3	≥ 3	4.1×10^{-12}
Taar7d	0	5	4.7×10^{-4}	0.2	≥ 3	1.1×10^{-13}
Taar7e				0.2	≥ 3	9.5×10^{-13}
Taar7f				0.2	≥ 3	7.3×10^{-14}
Taar8a				5.7	≥ 3	1
Taar8b	0	10	1.7×10^{-7}	0.7	≥ 3	1.8×10^{-7}
Taar8c				2.4	≥ 3	0.140657
Taar9	0	10	9.3×10^{-8}	0.3	≥ 3	1.2×10^{-11}
Olfr1507	117.4 ± 14.4	8	0.429866	117.6	≥ 3	1
Olfr578	79.4 ± 11.8	10	0.192455	116.4	≥ 3	1

Supplementary Table 8. List of *in situ* probes.

***OR* genes**							
**Gene**	**Synonym**	**OR class**	**Zone**	**Accession #**	**Nucleotide position**	**Cross-hybridized genes**	**Reference**
**Mixed *OR* probes**							
Olfr644	MOR13-1	I	1.0	AY317765.1	1-942	*Olfr643*	
Olfr578	MOR7-1	I	1.0	NM_147115	4-790	-	
Olfr1019	MOR180-1	II	1.0	AY318214.1	315-864	-	
Olfr1034	MOR227-8P, MOR245-14P, Olfr1533-ps1	II	1.0	NM_001011872.2	1-1013	*Olfr1036*	
Olfr145	MOR161-6, K21	II	1.0	NM_146313.1	1-930	*Olfr874, Olfr875, Olfr876*	
Olfr1395	MOR277-1	II	2.0	NM_146877.1	420-900	-	
Olfr2	MOR103-15, I7	II	4.0	AY317835.1	163-665	-	
Olfr1507	MOR28, MOR244-1	II	5.0	AY318726.1	1-899	*Olfr1508*	
**Mixed class I *OR* probes**							
Olfr543	MOR42-2	I	1.0	NM_001011782	38-987	-	
Olfr547	MOR31-4	I	1.0	NM_147079	31-910	-	
Olfr552	MOR28-1	I	1.0	NM_147102	15-927	-	
Olfr639	MOR12-1	I	1.0	NM_147084	1-922	-	Tetsuo Iwata, et al., (2017).
Olfr653	MOR33-1	I	1.0	NM_147074	32-960	-	
Olfr672	MOR32-4	I	1.0	NM_146760	9-919	-	
Olfr692	MOR36-1	I	1.0	NM_146355	23-952	-	
Olfr690	MOR31-2, Ors18	I	1.0	NM_020290	8-1002	-	

***Taar* genes (mixed *Taar* probes are composed of *Taar2*, *Taar3*, *Taar4*, *Taar5*, *Taar6*, *Taar7s*, *Taar8s*, and *Taar9*)**			
**Gene**	**Zone (dorsal or ventral)**	**Accession #**	**Nucleotide position**
Taar2	Dorsal	NM_001007266.1	29-996
Taar3	Dorsal	NM_001008429.1	21-1009
Taar4	Dorsal	NM_001008499.1	47-1041
Taar5	Dorsal	NM_001009574.1	25-988
Taar6	Both dorsal and ventral	NM_001010828.1	33-1027
Taar7a	Ventral	NM_001010829.1	3' UTR
Taar7b	Ventral	NM_001010827.1	3' UTR
Taar7d	Both dorsal and ventral	NM_001010838.1	3' UTR
Taar7e	Dorsal	NM_001010835.1	3' UTR
Taar7f	Dorsal	NM_001010839.1	3' UTR
Taar7s	Both dorsal and ventral	NM_001010829.1	78-934
Taar8s	Dorsal	NM_001010830.1	34-925
Taar9	Dorsal	NM_001010831.1	91-1004
**Other genes**			
GFP	-	U55762	679-1398

Supplementary Table 9. List of primers for mouse genotyping and *in situ* probe production.

**Target**	**Forward primer sequence**	**Reverse primer sequence**	**Product size [bp]**
		**Mouse genotyping**	
Taar5-ires-cre mouse	GCTAGAAGAGATGCCTTGTGTG	GCAAACCTTAGACTTCTCCGAC	761 (WT)
	CGTAACCTGGATAGTGAAACAGG	GCAAACCTTAGACTTCTCCGAC	427 (Cre)
Taar6-ires-cre mouse	GGAATTATCCAGTGCCCTCAAC	GTATCTGTGGTGCTTCAGGAGAC	846 (WT)
	CGTAACCTGGATAGTGAAACAGG	GTATCTGTGGTGCTTCAGGAGAC	486 (Cre)
TAAR enhancer 1 knockout mouse	AGCTGGGGATACAATGGTTG	GTTGTGAGCAAAACTAGGGTC	591 (WT)
	AGCTGGGGATACAATGGTTG	GTTGACCACATGCTATGAACC	355 (KO)
TAAR enhancer 2 knockout mouse	AGCAGACAAACTTACCCCTTC	GATCCACCTGTCCTTATGCTC	353 (WT)
	AGCAGACAAACTTACCCCTTC	GTGAGTTTGAGACCACCTTAGC	439 (KO)
TAAR enhancer 1 & 2 double knockout mouse			
TAAR enhancer 1	AGCTGGGGATACAATGGTTG	GTTGTGAGCAAAACTAGGGTC	591 (WT)
	AGCTGGGGATACAATGGTTG	GCAGTGTTCATTTTACAGGTGG	312 (KO)
TAAR enhancer 2	AGCAGACAAACTTACCCCTTC	GATCCACCTGTCCTTATGCTC	353 (WT)
	AGCAGACAAACTTACCCCTTC	GTGAGTTTGAGACCACCTTAGC	439 (KO)
TAAR enhancer 1-GFP transgenic mouse	GTGAGTCAAAATGACGCATG	GTTGTGAGCAAAACTAGGGTC	379
TAAR enhancer 2-tdTomato transgenic mouse	GTGAGTCAAAATGACGCATG	GTCATAAACCCACGGGCATC	432
Taar2-9 cluster knockout mouse	CAGCTAAGCACCACTACCAC	GAATGAGGATGTTGGTCAATG	508 (WT)
	CAGCTAAGCACCACTACCAC	GGAGTTATCACAGAGCTTCAGTCGAG	702 (KO)
lox-ZsGreen mouse	AAGGGAGCTGCAGTGGAGTA	CCGAAAATCTGTGGGAAGTC	297 (WT)
	GGCATTAAAGCAGCGTATCC	AACCAGAAGTGGCACCTGAC	199 (KI)
		***In situ* probe production**	
Taar2	CCTTTGATTGTTCAGAGTACGG	CCTTTGATTGTTCAGAGTACGG	968
Taar3	CGAAGACTTATCCAGCTGTC	TTGCAGTGTCTGAATTAGAGC	989
Taar4	TTGCTGCTGCAAACAGTTCT	AGGATGTGCAGGATGCAG	995
Taar5	TCTGGAGAGCAGCCTACA	GTGTCCTTGGTGAGAAGATC	964
Taar6	GCAGCTCTGCTATGAGAATGT	CAGAGAACAAGTTCATAGTGGCT	995
Taar7a	GATTCTTGGTTTAGTTGGGG	TGTAAATCTTGAATGGGTC	656
Taar7b	GATTCTTGGTTTAGTTGGGGA	GCACACCTTTGAAAACTTC	1049
Taar7d	GATTCTTGGTTTAGTCGGGG	CTACTGAGCTACCTCTTCAG	1044
Taar7e	TTTCTCTTGCTCCCAGGTTCTC	GTGCATATCTTTGAACACTTC	704
Taar7f	ACTCTGTCAACTGAGGCTCAG	CAAGAGCAAATTATACAGGG	500
Taar7s	AGAGCTGTGCTATGAGAACC	CTAGGATTTCATACACATACGTGG	857
Taar8s	CTCTGCTATGAGAACACGAATG	GGTTCATGGCTGAGTTATAATAGG	892
Taar9	CCTCGAGCCATCCTCTATG	GAATCAGCCCTGAAGACTTTGC	914
Olfr644	ATGGGAGGTGAAGCCCACAA	AAATTTGTTCCCCAGCAGGA	942
Olfr578	GCCATCCTTTACAACAGCAGC	GGTTCATGGCTGAGTTATAATAGG	785
Olfr1019	AGGCTTTGTGGATGCTGAGT	CAAGGGGTTCAGCATAGGAA	550

Olfr1034	CGTTTTTCCTTGCAGAAAAC	ACACATTTGGATAAACACGC	1013
Olfr145	ATGGCCACTGAAAATGCCTC	GGAAAATGAACTTCTGCTCA	930
Olfr1395	TGTCTGCTGGCTCATGGTAG	TCCGGTCACTTCTTTGTTCC	481
Olfr2	ACCCTCCACAAACCCATGTA	GAAGCCCCAGTGACAGAGAG	503
Olfr1507	ATGGAAAAGGCTGTCCTCA	GCACTCTTCATGTCCTCATTC	899
Olfr543	GGTGAGGATGGAAATACCAGTATC	GGTGACTGCATTCCACAGAG	950
Olfr547	CACACAGTCTTCCATTTGTTGG	GCTCCTGGATTTGCTTGGT	880
Olfr552	TATCACCTCAACCCACCCAG	GAATAGCCCTAAGACTCGCTCT	913
Olfr639	ATGGGAGCAGAGAACAATGAAAGTC	AGTTGGCAATTCCCTGTTGG	922
Olfr653	TCTCTGCTGCTATCCCCAATG	GTGGAGATTATTGTCACCTTCTCTTTAC	929
Olfr672	AGGCAACAGCTCATGGATTCA	TATCACCACACGATCTCGAATCTG	911
Olfr692	ACAGAATGGCAGAAAGTTCGCA	ATCGGTCCCTAATCTGCTTGG	930
Olfr690	TTTGACCAGACCTCACAGCTG	ATGCTGTCCCTAATTTGCTTGG	995
GFP	ATGGTGAGCAAGGGCGA	TTACTTGTACAGCTCGTCCATGC	720
Degenerate OR	GCITA(C/T)GA(C/T)CGITA(C/T)GTIGCIA TITG	ACIACIGAIAG(G/A)TGIGAI(G/C)C(G/A) CAIGT	327

Supplementary Table10. List of plasmid information.

**plasmid**	**Addgene #**	**Usage**
PBCAG-eGFP	40973	Modified this plasmid for PiggyBac transposon system
pX330-U6-Chimeric_BB-CBh-hSpCas9	42230	sgRNA template
pX260-U6-DR-BB-DR-Cbh-NLS-hSpCas9-NLS-H1-shorttracr-PGK-puro	42229	SpCas9 template

Supplementary Figure 1. Representative FACS density plots. (a) Representative plots with gates showing sorted ZsGreen-positive and ZsGreen-negative cells in *Taar5-ires-Cre*; *lox-ZsGreen* mice. (b) The classical olfactory chaperon molecules (*Rtp1* and *Rtp2*) were expressed at similar level in reporter-positive (*Taar5*+ or *Taar6*+) and reporter-negative (control) cells (n = 10 for control cells, n = 7 for *Taar5*+ reporter-positive cells, n = 1 for *Taar6*+ reporter-positive cells). Data are presented as mean values ± SEM.

Supplementary Figure 2. Similar chromatin accessibility of canonical signaling molecules between TAAR OSNs and OMP-positive OSNs. ATAC-seq signals plotted across different genomic regions containing various olfactory signaling molecules. ATAC-seq signals were normalized to median peak values of each sample. Below the signal tracks, exons of genes were depicted. Arrows inside exons indicate direction of sense strand. The NM_010307 isoform of *Gnal* gene is possibly the major variant expressed in the MOE based on the fact that ATAC-seq peaks are found in its promoter region but not the NM_177137 isoform. This is consistent with our RNA-seq data.

Supplementary Figure 3. The *Taar* genes were downregulated in mature OSNs when Lhx2 or Ldb1 is deleted. (a) Published RNA-seq data of 5 mature OSN samples from control mice, 3 mature OSN samples from Lhx2 knockout mice, and 4 mature OSN samples from Ldb1 knockout mice were retrieved from the GEO (GSE112153) [17] and analyzed to examine changes of the *Taar* gene expression. Normalized counts of the 14 olfactory *Taar* genes were depicted in control mice, Lhx2 knockout mice, and Ldb1 knockout mice. Data are presented as mean values ± SEM. (b, c) Multiple alignment of TAAR enhancer 1 (b) and TAAR enhancer 2 (c) obtained from 8 placental mammals. Motif analyses in the mouse sequences revealed 2 Lhx2-binding motifs (pink box) and 1 Lhx2/Ebf composite motif (light purple box) in TAAR enhancer 1 (b), and 2 Lhx2-binding motifs, 1 Ebf-binding motif (light green box), and 1 Lhx2/Ebf composite motif in TAAR enhancer 2 (c). The orientation of the motifs is represented by arrows pointing in different directions. Note that there are duplications of TAAR enhancer

2 in cow and horse. In a, n.s. not significant, * $p < 0.05$, ** $p < 0.01$, *** $p < 0.001$, by the Wald test (two-sided) in DESeq2 package.

Supplementary Figure 4. Conservation analysis of the two TAAR enhancers. (a) Summary of 8 Glires, 21 Euarchontoglires, 40 placental mammals, and 60 vertebrates included in the conservation analysis in Figure 3a. The subset information and evolutionary relationships were obtained from UCSC. (b) VISTA nucleotide percent identity plots of the mouse *Taar* cluster ranging from *Taar1* to *Taar7a* compared with that in orangutan, gibbon, dolphin, sperm whale, seal, sea lion, and dog. (c) VISTA nucleotide percent identity plots of mouse TAAR enhancer 2 and two adjacent *Taar* genes compared with that in in cow, sheep, and horse. The arrows indicate the transcriptional orientations of *Taar* genes in mouse genome. Conservation between 50% and 100% are shown as peaks. Highly conserved regions (> 100 bp width and > 70% identity) in exons, intergenic regions, and TAAR enhancers are shown in blue, red, and cyan, respectively.

Supplementary Figure 5. Downregulation of specific *Taar* genes by TAAR enhancer 1 deletion. (a) The 14 DEGs were identified using the criteria of $q < 0.05$ and fold change > 1.5-fold in RNA-seq data of homozygous TAAR enhancer 1 knockout mice and wild type mice. The 9 *Taar* genes that showed significant changes are indicated by blue dots. The 5 other DEGs are indicated by red dots. And other *Taar* genes are indicated by blue circles. (b) Normalized counts of the 14 olfactory *Taar* genes from RNA-seq data were depicted in wild type (+/+), heterozygous (+/-), and homozygous (-/-) TAAR enhancer 1 knockout mice ($n = 3$). Data are presented as mean values ± SEM. (c) Normalized counts of the *OR* genes from RNA-seq data were depicted in wild type (+/+), heterozygous (+/-), and homozygous (-/-) TAAR enhancer 1 knockout mice. (d) Top, representative confocal images of immunohistochemistry staining for TAAR6 and Olfr1507 in wild type (+/+), heterozygous (+/-), and homozygous (-/-) TAAR enhancer 1 knockout mice. Blue fluorescence represented DAPI counterstaining. Scale bar = 25 μm. Bottom, percentage of positive cell numbers in

heterozygous (+/-) or homozygous (-/-) mice compared to wild type (+/+) was plotted. Data are presented as mean values ± SEM (n = 7 for TAAR6 staining, n = 5 for Olfr1507 staining). (e) Left, representative confocal images of immunohistochemistry staining for Caspase-3 in wild type (+/+), heterozygous (+/-), and homozygous (-/-) TAAR enhancer 1 knockout mice. Blue fluorescence represented DAPI counterstaining. Scale bar = 25 μm. Right, percentage of positive cell numbers in heterozygous (+/-) or homozygous (-/-) mice compared to wild type (+/+) was plotted. Data are presented as mean values ± SEM (n = 6). In b, ** $p < 0.01$, *** $p < 0.001$, by the LRT and Wald test (two-sided) in DESeq2 package.

Supplementary Figure 6. Downregulation of specific *Taar* genes by TAAR enhancer 2 deletion. (a) The 6 DEGs were identified using the criteria of $q < 0.05$ and fold change > 1.5-fold in RNA-seq data of homozygous TAAR enhancer 2 knockout mice and wild type mice. The 5 *Taar* genes that showed significant changes are indicated by blue dots. Another DEG is indicated by red dots. All of the other *Taar* genes are indicated by blue circles. (b) Normalized counts of the 14 olfactory *Taar* genes from RNA-seq data were depicted in wild type (+/+), heterozygous (+/-), and homozygous (-/-) TAAR enhancer 2 knockout mice (n = 3). Data are presented as mean values ± SEM. (c) Normalized counts of the *OR* genes from RNA-seq data were depicted in wild type (+/+), heterozygous (+/-), and homozygous (-/-) TAAR enhancer 2 knockout mice (n = 3). (d) Correlation between log2-fold change values of positive cell numbers by *in situ* hybridization (Y-axis, $n \geq 3$) and gene expression by RNA-seq (X-axis, n = 3) for the *Taar* genes. Pearson correlation coefficient $r = 0.88$ (two-sided, no adjustments were made), $p = 0.0001$. (e) Left, representative images of immunohistochemistry staining for TAAR5 and Olfr552 in wild type (+/+), heterozygous (+/-), and homozygous (-/-) TAAR enhancer 2 knockout mice. Blue fluorescence represented DAPI counterstaining. Scale bar = 25 μm. Right, percentage of positive cell numbers in heterozygous (+/-) or homozygous (-/-) mice compared to wild type (+/+) was plotted. Data are presented as mean values ± SEM (n = 5). (f) Left, representative images of immunohistochemistry staining for Caspase-3 in wild

type (+/+), heterozygous (+/-), and homozygous (-/-) TAAR enhancer 2 knockout mice. Blue fluorescence represented DAPI counterstaining. Scale bar = 25 μm. Right, percentage of positive cell numbers in heterozygous (+/-) or homozygous (-/-) mice compared to wild type (+/+) was plotted. Data are presented as mean values ± SEM (n = 5). In b, * $p < 0.05$, ** $p < 0.01$, *** $p < 0.001$, by the LRT and Wald test (two-sided) in DESeq2 package. In e, * $p < 0.05$, *** $p < 0.001$, by one-way ANOVA and post-hoc Tukey's test.

Supplementary Figure 7. Loss of *Taar* gene expression by deletion of the two TAAR enhancers. (a) The 13 DEGs were identified using the criteria of $q < 0.05$ and fold change > 1.5-fold in RNA-seq data of homozygous TAAR enhancer 1 & 2 double knockout mice and wild type mice. The 12 *Taar* genes that showed significant changes are indicated by blue dots. Another DEG is indicated by red dots. All of the other *Taar* genes are indicated by blue circles. (b) Normalized counts of the 14 olfactory *Taar* genes from RNA-seq data were depicted in wild type (+/+), heterozygous (+/-), and homozygous (-/-) TAAR enhancer 1 & 2 double knockout mice ($n \geq 3$). Data are presented as mean values ± SEM. (c) Normalized counts of the *OR* genes from RNA-seq data were depicted in wild type (+/+), heterozygous (+/-), and homozygous (-/-) TAAR enhancer 1 & 2 double knockout mice. (d) Left, representative images of immunohistochemistry staining for Caspase-3 in wild type (+/+), heterozygous (+/-), and homozygous (-/-) TAAR enhancer 1 & 2 double knockout mice. Blue fluorescence represented DAPI counterstaining. Scale bar = 25 μm. Right, percentage of positive cell numbers in heterozygous (+/-) or homozygous (-/-) mice compared to wild type (+/+) was plotted. Data are presented as mean values ± SEM (n = 5). In b, *** $p < 0.001$, by the LRT and Wald test (two-sided) in DESeq2 package.

Supplementary Figure 8. Functional analysis of TAAR enhancers in zebrafish and mouse. (a) Schematic depiction of the E1b reporter expression construct used in zebrafish embryo injection. Various enhancer candidates were amplified from mouse genomic DNA and

fused to the E1b minimal promoter followed by the GFP sequence. (b) Representative bright field (left) and fluorescent images of injected zebrafish larvae head at 4 dpf (days post-fertilization). (c) Percentage of injected zebrafish embryos with GFP-positive OSNs at 24-48 hpf (hours post-fertilization) for the TAAR enhancers or the Sifnos OR enhancer that served as a positive control. (d) Images of the MOE sections from TAAR enhancer 1-GFP (left) or TAAR enhancer 2-tdTomato mice (right). To better show the reporter-positive cells, the positions of reporter-positive OSNs are highlighted with white dots. Insets show higher magnification of reporter-positive OSNs ($n \geq 3$). Scale bar = 200 μm. (e) Whole-mount fluorescent images of the medial view of olfactory bulb in TAAR enhancer 1-GFP; TAAR enhancer 2-tdTomato transgenic mice. A, anterior; P, posterior; D, dorsal; V, ventral ($n \geq 3$). Scale bar = 200 μm.

Supplementary Figure 9. The TAAR OSN-specific enhancer activity of the two TAAR enhancers. (a) FACS-sorted reporter-positive (GFP+ in TAAR enhancer 1-GFP mice or tdTomato+ in TAAR enhancer 2-tdTomato mice) and reporter-negative (GFP- in TAAR enhancer 1-GFP mice or tdTomato- in TAAR enhancer 2-tdTomato mice) cells were collected for RNA-seq experiments. The TPM values extracted for the total *Taar* and *OR* genes were plotted. (b) The TPM values of all the 15 *Taar* genes were plotted in reporter-positive (GFP+ or tdTomato+) and reporter-negative (GFP- or tdTomato-) cells, showing that reporter-positive cells had higher *Taar* gene expression than reporter-negative cells. (c) Top left, confocal images of *in situ* hybridization of individual probes of *Taar* genes (magenta) and the following immunohistochemistry by the GFP antibody (green) in a TAAR enhancer 1-GFP transgenic mouse. Top right, confocal images of *in situ* hybridization of individual probes of *Taar* genes (green) and the following immunohistochemistry by the tdTomat antibody (magenta) in a TAAR enhancer 2-tdTomato transgenic mouse. Scale bar = 25 μm. Bottom, bar plots showing the percentages of GFP-positive cells (left) or tdTomato-positive cells (right) that were co-labeled with individual *Taar* probes ($n \geq 3$).

Supplementary Figure 10. Validation of *Taar2-9* cluster knockout mice. (a) Normalized counts of the 14 olfactory *Taar* genes and the *OR* genes from RNA-seq data were depicted in wild type (+/+), heterozygous (+/-), and homozygous (-/-) *Taar2-9* cluster knockout mice. Expression of the olfactory *Taar* genes was abolished, while expression of the *OR* genes was not significantly changed (n ≥ 3). Data are presented as mean values ± SEM. (b) Representative images of *Taar4*, *Taar6*, and *Olfr77* expression in wild type (+/+), heterozygous (+/-), and homozygous (-/-) *Taar2-9* cluster knockout mice using single color *in situ* hybridization. Scale bar = 25 μm (n ≥ 3). (c) Representative confocal images of immunohistochemistry staining for TAAR4 in wild type (+/+), heterozygous (+/-), and homozygous (-/-) *Taar2-9* cluster knockout mice. Scale bar = 25 μm (n ≥ 3).

Supplementary Table 1. List of genome sequences used for VISTA plots. The information of extracted genome sequences from various mammalian species was listed.

Supplementary Table 2. *In situ* analysis in TAAR enhancer 1 knockout mice. Summary of quantification results (the number of sections analyzed, the average number of positive cells per section, percentage of expression, and statistics) for all of the functional *Taar* genes and 2 representative *OR* genes by *in situ* hybridization experiments. Percentage of expression was calculated by dividing the average number per section of heterozygous or homozygous TAAR enhancer 1 knockout mice by that of wild type littermates. p values were calculated by one-way ANOVA.

Supplementary Table 3. Comparison of *in situ* and RNA-seq analyses in TAAR enhancer 1 knockout mice. Summary of quantification results for all of the functional *Taar* genes and 2 representative *OR* genes by *in situ* hybridization and RNA-seq experiments. Percentage of expression for *in situ* was calculated by dividing the average number per section of

homozygous TAAR enhancer 1 knockout mice by that of wild type littermates. Percentage of expression for RNA-seq (Supplementary Data 1) was displayed on the right column (ratio of homozygous and wild type TAAR enhancer 1 knockout mice). p values were calculated by one-way ANOVA and post-hoc Tukey's test for *in situ*, and by the LRT and Wald test (two-sided) for RNA-seq.

Supplementary Table 4. *In situ* analysis in TAAR enhancer 2 knockout mice. Summary of quantification results (the number of sections analyzed, the average number of positive cells per section, percentage of expression, and statistics) for all of the functional *Taar* genes and 2 representative *OR* genes by *in situ* hybridization experiments. Percentage of expression was calculated by dividing the average number per section of heterozygous or homozygous TAAR enhancer 2 knockout mice by that of wild type littermates. p values were calculated by one-way ANOVA.

Supplementary Table 5. Comparison of *in situ* and RNA-seq analyses in TAAR enhancer 2 knockout mice. Summary of quantification results for all of the functional *Taar* genes and 2 representative *OR* genes by *in situ* hybridization and RNA-seq experiments. Percentage of expression for *in situ* was calculated by dividing the average number per section of homozygous TAAR enhancer 2 knockout mice by that of wild type littermates. Percentage of expression for RNA-seq (Supplementary Data 1) was displayed on the right column (ratio of homozygous and wild type TAAR enhancer 2 knockout mice). p values were calculated by one-way ANOVA and post-hoc Tukey's test for *in situ*, and by the LRT and Wald test (two-sided) for RNA-seq.

Supplementary Table 6. *In situ* analysis in TAAR enhancer 1 & 2 double knockout mice. Summary of quantification results (the number of sections analyzed, the average number of positive cells per section, percentage of expression, and statistics) for all of the functional *Taar* genes and 2 representative *OR* genes by *in situ* hybridization experiments. Percentage of

expression was calculated by dividing the average number per section of heterozygous or homozygous TAAR enhancer 1 & 2 double knockout mice by that of wild type littermates. p values were calculated by one-way ANOVA.

Supplementary Table 7. Comparison of *in situ* and RNA-seq analyses in TAAR enhancer 1 & 2 double knockout mice. Summary of quantification results for all of the functional *Taar* genes and 2 representative *OR* genes by *in situ* hybridization and RNA-seq experiments. Percentage of expression for *in situ* was calculated by dividing the average number per section of homozygous TAAR enhancer 1 & 2 double knockout mice by that of wild type littermates. Percentage of expression for RNA-seq (Supplementary Data 1) was displayed on the right column (ratio of homozygous and wild type TAAR enhancer 1 & 2 double knockout mice). p values were calculated by one-way ANOVA and post-hoc Tukey's test for *in situ*, and by the LRT and Wald test (two-sided) for RNA-seq.

Supplementary Table 8. List of *in situ* probes. Summary of probes used for *in situ* analyses of *OR*, *Taar*, and *GFP* mRNA expression. The cross-hybridized genes were listed if the percentage coverage and nucleotide identity were both above 80%.

Supplementary Table 9. List of primers for mouse genotyping and *in situ* probe production. Summary of primers used for mouse genotyping and *in situ* probe production. WT, wild type; KO, knockout; KI, knockin.

Supplementary Table 10. List of plasmid information. Summary of plasmids used for generating sgRNA, spCas9 mRNA, and PiggyBac transposon system.
