# Supplementary material for: Coordination of two enhancers drives expression of olfactory trace amine-associated receptors
Source: Nat Commun. 2021 Jun 18;12:3798. doi: 10.1038/s41467-021-23823-4 (PMC8213717; doi:10.1038/s41467-021-23823-4)
Supplement: Supplementary file 3 — Description of Additional Supplementary Files [file 41467_2021_23823_MOESM3_ESM.pdf]

Description of Additional Supplementary Files

File Name: Supplementary Data 1
Description: **Expression changes of all genes by RNA-seq analyses on TAAR enhancer knockout mice.** Summary of RNA-seq results of TAAR enhancer 1 knockout mice, TAAR enhancer 2 knockout mice, and TAAR enhancer 1 & 2 knockout mice.
