# Supplementary material for: Coordination of two enhancers drives expression of olfactory trace amine-associated receptors
Source: Nat Commun. 2021 Jun 18;12:3798. doi: 10.1038/s41467-021-23823-4 (PMC8213717; doi:10.1038/s41467-021-23823-4)
Supplement: Supplementary file 5 — Reporting Summary [file 41467_2021_23823_MOESM5_ESM.pdf]

nature research

Corresponding author(s): Qian Li

Last updated by author(s): Mar 11, 2021

Reporting Summary

Nature Research wishes to improve the reproducibility of the work that we publish. This form provides structure for consistency and transparency in reporting. For further information on Nature Research policies, see our Editorial Policies and the Editorial Policy Checklist.

Statistics

For all statistical analyses, confirm that the following items are present in the figure legend, table legend, main text, or Methods section.

n/a	Confirmed	
☐	☒	The exact sample size (*n*) for each experimental group/condition, given as a discrete number and unit of measurement
☒	☐	A statement on whether measurements were taken from distinct samples or whether the same sample was measured repeatedly
☐	☒	The statistical test(s) used AND whether they are one- or two-sided<br>*Only common tests should be described solely by name; describe more complex techniques in the Methods section.*
☒	☐	A description of all covariates tested
☒	☐	A description of any assumptions or corrections, such as tests of normality and adjustment for multiple comparisons
☐	☒	A full description of the statistical parameters including central tendency (e.g. means) or other basic estimates (e.g. regression coefficient) AND variation (e.g. standard deviation) or associated estimates of uncertainty (e.g. confidence intervals)
☐	☒	For null hypothesis testing, the test statistic (e.g. *F*, *t*, *r*) with confidence intervals, effect sizes, degrees of freedom and *P* value noted<br>*Give P values as exact values whenever suitable.*
☒	☐	For Bayesian analysis, information on the choice of priors and Markov chain Monte Carlo settings
☒	☐	For hierarchical and complex designs, identification of the appropriate level for tests and full reporting of outcomes
☐	☒	Estimates of effect sizes (e.g. Cohen's *d*, Pearson's *r*), indicating how they were calculated

Our web collection on statistics for biologists contains articles on many of the points above.

Software and code

Policy information about availability of computer code

Data collection	Images from in situ and immunohistochemistry experiments were taken using a Leica TCS SP8 confocal microscope. Whole-mount images were taken by a Nikon Ti2-E&CSU-W1 confocal microscope.
Data analysis	The following software was used:<br>Bowtie 2 version 2.2.9<br>MACS2 version 2.1.1.20160309<br>DiffBind version 2.8.0<br>Hisat2 version 2.1.0<br>Cufflinks version 2.2.1<br>Subread package version 1.6.2<br>DESeq2 version 1.20.0<br>Bedtools version 2.27.1<br>HOMER version 4.9<br>R version 3.5.1<br>GraphPad Prism version 8.4.0 (455)<br>Web-based VISTA program version 2.0 (http://genome.lbl.gov/vista/mvista/submit.shtml)<br>blastn algorithm version 2.11.0 (https://blast.ncbi.nlm.nih.gov/Blast.cgi?PROGRAM=blastn&PAGE_TYPE=BlastSearch&LINK_LOC=blasthome)

For manuscripts utilizing custom algorithms or software that are central to the research but not yet described in published literature, software must be made available to editors and reviewers. We strongly encourage code deposition in a community repository (e.g. GitHub). See the Nature Research guidelines for submitting code & software for further information.

Data

Policy information about availability of data

All manuscripts must include a data availability statement. This statement should provide the following information, where applicable:

- Accession codes, unique identifiers, or web links for publicly available datasets
- A list of figures that have associated raw data
- A description of any restrictions on data availability

The data supporting the findings of this study are included within the article and its Supplemental files. Reagents are available from the corresponding author upon reasonable request. The source data underlying Figure 1B-D, 2D, 2F, 4F-H, 5F, 6F, 7C, 7E, 8C, and Supplementary Figure 1B, 3A, 5D, 5E, 6E, 6F, 7D, 8C, 9A-C are provided as a Source Data file.
The raw and processed sequencing data were deposited in the Gene Expression Omnibus with the accession number GSE163778 (https://www.ncbi.nlm.nih.gov/geo/query/acc.cgi?acc=GSE163778).

Field-specific reporting

Please select the one below that is the best fit for your research. If you are not sure, read the appropriate sections before making your selection.

- [x] Life sciences
- [ ] Behavioural & social sciences
- [ ] Ecological, evolutionary & environmental sciences

For a reference copy of the document with all sections, see nature.com/documents/nr-reporting-summary-flat.pdf

Life sciences study design

All studies must disclose on these points even when the disclosure is negative.

Sample size	A sample size of at least three independent biological replicates was selected for RNA-seq, in situ, and immunohistochemistry experiments in the figures. This size was selected because multiple statistical approaches have been developed to allow identification of significantly changed genes from more than two biological replicates of high throughput sequencing data (e.g. DEseq2) and experimental data (e.g. one-way ANOVA). Wherever possible, additional biological replicates were included. Sample sizes were based on previous publications with similar experimental systems (Tetsuo Iwata et al., Nature Communications, 2017).
Data exclusions	For in situ and immunohistochemistry expeiments, the damaged tissues were excluded from the imaging and quantification. This exclusion was pre-established. No other data were excluded.
Replication	At least three independent biological replicates were performed for each experiment. Replicate experiments yielded the same results.
Randomization	No experiments were performed with live animals. For the purpose of sorting reporter-positive and reporter-negative cells, animals of similar age were grouped by genotype and sorted together.
Blinding	Animals were used as the source of tissue and primary cells, so knowledge of genotype was required for proper handling and cell sorting. Blinding was not attempted for experiments performed on sorted cells.

Reporting for specific materials, systems and methods

We require information from authors about some types of materials, experimental systems and methods used in many studies. Here, indicate whether each material, system or method listed is relevant to your study. If you are not sure if a list item applies to your research, read the appropriate section before selecting a response.

Materials & experimental systems

n/a	Involved in the study	
[ ]	[x]	Antibodies
[x]	[ ]	Eukaryotic cell lines
[x]	[ ]	Palaeontology and archaeology
[ ]	[x]	Animals and other organisms
[x]	[ ]	Human research participants
[x]	[ ]	Clinical data
[x]	[ ]	Dual use research of concern

Methods

n/a	Involved in the study	
[ ]	[x]	ChIP-seq
[ ]	[x]	Flow cytometry
[x]	[ ]	MRI-based neuroimaging

Antibodies

Antibodies used	Primary antibodies including TAAR4, TAAR5, TAAR6 (homemade, reference: Johnson et al., 2012, PNAS), caspase-3 (Cell Signaling, 9661), GFP (Abcam, ab13970), and tdTomato (Takara, 632496) were used at 1:5,000, 1:5,000, 1:1,000, 1:500, 1:1,000, and 1:500 dilution. Secondary antibodies including Donkey Anti-Chicken IgY conjugated to Alexa Fluo 488 (Jackson

ImmunoResearch,703-545-155), Donkey Anti-Rabbit IgG conjugated to Alexa Fluo 488 (Jackson ImmunoResearch, 711-545-152), and Donkey Anti-Guinea Pig IgG conjugated to Cy3 (Jackson ImmunoResearch, 706-165-148) were used at 1:1,000 dilution. H3K9me3 antibody (ABclonal, A2360) was used for ChIP-seq.

Validation

TAAR4, TAAR5, TAAR6 - the three antibodies have been validated and stated in Johnson et al., PNAS, 2012.
Caspase-3 - Manufacturer states that the antibody is validated for immunofluorescence in Staurosporine treated HT-29 cells.
GFP - Manufacturer states that the antibody is validated for immunofluorescence in GFP-transfected NIH3T3 cells.
tdTomato - Manufacturer states that the antibody is validated for immunofluorescence in previous publication (Jinfei D Ni et al., eLife, 2019).
H3K9me3 - Manufacturer states that the antibody is validated for ChIP in 293T cells.
Donkey Anti-Chicken IgY conjugated to Alexa Fluo 488 - Manufacturer states that antibody is validated for immunofluorescence.
Donkey Anti-Rabbit IgG conjugated to Alexa Fluo 488 - Manufacturer states that antibody is validated for immunofluorescence.
Donkey Anti-Guinea Pig IgG conjugated to Cy3 - Manufacturer states that antibody is validated for immunofluorescence.

Animals and other organisms

Policy information about studies involving animals; ARRIVE guidelines recommended for reporting animal research

Laboratory animals

The information about the zebrafish used in this study were:
species: Danio rerio
strain: AB with mitfa +/- or -/-
age: larval/embryonic stage < 7 days post fertilization.
This study used several mouse lines on mixed C57BL/6J and 129 backgrounds. Experimental genotypes were:
Omp-ires-GFP - 6-8 weeks old, male and female mice were used
Taar5-ires-Cre; lox-L10-GFP - 6-8 weeks old, male and female mice were used
Taar5-ires-Cre; lox-ZsGreen - 6-8 weeks old, male and female mice were used
Taar6-ires-Cre; lox-ZsGreen - 6-8 weeks old, male and female mice were used
TAAR enhancer 1 knockout - 6-8 weeks old, male and female mice were used
TAAR enhancer 2 knockout - 14 days postnatal , male and female mice were used
TAAR enhancer 1 & 2 knockout - 14 days postnatal , male and female mice were used
Taar2-9 cluster knockout - 14 days postnatal , male and female mice were used
TAAR enhaner 1-GFP transgenic - 14 days postnatal , male and female mice were used
TAAR enhancer 2-tdTomato transgenic - 14-21 days postnatal , male and female mice were used

Wild animals

Study did not involve wild animals.

Field-collected samples

Study did not involve specimens collected from the field.

Ethics oversight

All mouse experiments were approved by the Animal Ethics Committee of Shanghai Jiao Tong University School of Medicine and the Institutional Animal Care and Use Committee (Department of Laboratory Animal Science, Shanghai Jiao Tong University School of Medicine, animal protocol number A-2016-049).

Note that full information on the approval of the study protocol must also be provided in the manuscript.

ChIP-seq

Data deposition

☒ Confirm that both raw and final processed data have been deposited in a public database such as GEO.

☒ Confirm that you have deposited or provided access to graph files (e.g. BED files) for the called peaks.

Data access links
May remain private before publication.

The raw and processed ChIP-seq data were deposited in the Gene Expression Omnibus with the accession number GSE163778 (https://www.ncbi.nlm.nih.gov/geo/query/acc.cgi?acc=GSE163778).

Files in database submission

GFP-negative_TAAR5-Cre&L10-GFP_H3K9me3_Input-1.R1.fastq.gz
GFP-negative_TAAR5-Cre&L10-GFP_H3K9me3_Input-1.R2.fastq.gz
GFP-negative_TAAR5-Cre&L10-GFP_H3K9me3_Input-2.R1.fastq.gz
GFP-negative_TAAR5-Cre&L10-GFP_H3K9me3_Input-2.R2.fastq.gz
GFP-negative_TAAR5-Cre&L10-GFP_H3K9me3_ChIPseq-1.R1.fastq.gz
GFP-negative_TAAR5-Cre&L10-GFP_H3K9me3_ChIPseq-1.R2.fastq.gz
GFP-negative_TAAR5-Cre&L10-GFP_H3K9me3_ChIPseq-2.R1.fastq.gz
GFP-negative_TAAR5-Cre&L10-GFP_H3K9me3_ChIPseq-2.R2.fastq.gz
GFP-positive_TAAR5-Cre&L10-GFP_H3K9me3_Input-1.R1.fastq.gz
GFP-positive_TAAR5-Cre&L10-GFP_H3K9me3_Input-1.R2.fastq.gz
GFP-positive_TAAR5-Cre&L10-GFP_H3K9me3_Input-2.R1.fastq.gz
GFP-positive_TAAR5-Cre&L10-GFP_H3K9me3_Input-2.R2.fastq.gz
GFP-positive_TAAR5-Cre&L10-GFP_H3K9me3_ChIP-1.R1.fastq.gz
GFP-positive_TAAR5-Cre&L10-GFP_H3K9me3_ChIP-1.R2.fastq.gz
GFP-positive_TAAR5-Cre&L10-GFP_H3K9me3_ChIP-2.R1.fastq.gz
GFP-positive_TAAR5-Cre&L10-GFP_H3K9me3_ChIP-2.R2.fastq.gz

GFP-negative_TAAR5-Cre&L10-GFP_H3K9me3_Input.bw
GFP-negative_TAAR5-Cre&L10-GFP_H3K9me3_ChIPseq.bw
GFP-positive_TAAR5-Cre&L10-GFP_H3K9me3_Input.bw
GFP-positive_TAAR5-Cre&L10-GFP_H3K9me3_ChIPseq.bw

Genome browser session (e.g. UCSC)

http://www.genome.ucsc.edu/cgi-bin/hgTracks?db=mm10&lastVirtModeType=default&lastVirtModeExtraState=&virtModeType=default&virtMode=0&nonVirtPosition=&position=chr10%3A23860857%2D24061380&hgsid=1005639891_wA6J9KHwK0ZJacBktha1hSs6AYi2

Methodology

Replicates	No replicates were performed.
Sequencing depth	Each sample had ~ 5 million paired-end 75 bp reads, ~ 4 million of which were uniquely mappable.
Antibodies	H3K9me3 antibody (ABclonal, A2360)
Peak calling parameters	No peaks were called.
Data quality	Data had high mapping rate, and showed strong H3K9me3 enrichment around OR genes as other labs previously reported.
Software	Reads were mapped to the mouse reference genome (mm10) with Bowtie 2 (version 2.2.9) using the default parameters.

Flow Cytometry

Plots

Confirm that:

- [x] The axis labels state the marker and fluorochrome used (e.g. CD4-FITC).
- [x] The axis scales are clearly visible. Include numbers along axes only for bottom left plot of group (a 'group' is an analysis of identical markers).
- [x] All plots are contour plots with outliers or pseudocolor plots.
- [ ] A numerical value for number of cells or percentage (with statistics) is provided.

Methodology

Sample preparation	Mice were sacrificed with CO_2 followed by cervical dislocation. The MOE tissue was dissected and cells were dissociated using Papain Dissociation System (Worthington Biochemical) following manufacturer's instructions with minor modifications. Briefly, dissociation reaction was incubated at 37C for 15 minutes. The tissue was triturated for 10-15 times with a cut P1000 pipette tip. Cells were then filtered by 40m strainer (Falcon) and centrifuged at 400 g for 2 minutes. Cell pellets were resuspended in DMEM (Gibco) and kept on ice for sorting. OSNs were sorted on a FACSJazz Cell Sorter (BD) or MoFlo Astrios EQ (Beckman Coulter) with a 488-nm or 561-nm laser.
Instrument	FACSJazz Cell Sorter (BD) or MoFlo Astrios EQ (Beckman Coulter)
Software	No post-sort analysis of flow data was performed
Cell population abundance	Post-sorted cells were initially checked for presence of the fluorescent reporters. Purities of greater than 95% were routinely observed.
Gating strategy	Unlabeled negative control population were analyzed to establish gate positions.

- [x] Tick this box to confirm that a figure exemplifying the gating strategy is provided in the Supplementary Information.
